# Identification of candidate diagnostic biomarkers for adolescent idiopathic scoliosis using UPLC/QTOF-MS analysis: a first report of lipid metabolism profiles

**DOI:** 10.1038/srep22274

**Published:** 2016-03-01

**Authors:** Zhi-jian Sun, Hong-mei Jia, Gui-xing Qiu, Chao Zhou, Shigong Guo, Jian-guo Zhang, Jian-xiong Shen, Yu Zhao, Zhong-mei Zou

**Affiliations:** 1 Department of Orthopaedics, Peking Union Medical College Hospital, Chinese Academy of Medical Sciences and Peking Union Medical College, Dongcheng District Shuaifuyuan No. 1, Beijing 100730, China; 2 Institute of Medicinal Plant Development, Chinese Academy of Medical Sciences and Peking Union Medical College, No. 151 Malianwa North Road, Haidian District, Beijing 100193, China; 3 Department of Trauma & Orthopaedic Surgery, Lister Hospital, Stevenage, UK

## Abstract

Adolescent idiopathic scoliosis (AIS) is a complex spine deformity, affecting approximately 1–3% adolescents. Earlier diagnosis could increase the likelihood of successful conservative treatment and hence reduce the need for surgical intervention. We conducted a serum metabonomic study to explore the potential biomarkers of AIS for early diagnosis. Serum metabolic profiles were firstly explored between 30 AIS patients and 31 healthy controls by ultra high-performance liquid chromatography coupled with quadrupole time-of-flight mass spectrometry. Then, the candidate metabolites were validated in an independent cohort including 31 AIS patients and 44 controls. The results showed that metabolic profiles of AIS patients generally deviated from healthy controls in both the discovery set and replication set. Seven differential metabolites were identified as candidate diagnostic biomarkers, including PC(20:4), 2-hexenoylcarnitine, beta-D-glucopyranuronicacid, DG(38:9), MG(20:3), LysoPC(18:2) and LysoPC(16:0). These candidate metabolites indicated disrupted lipid metabolism in AIS, including glycerophospholipid, glycerolipid and fatty acid metabolism. Elevated expressions of adipose triglyceride lipase and hormone sensitive lipase in adipose tissue further corroborated our findings of increased lipid metabolism in AIS. Our findings suggest that differential metabolites discovered in AIS could be used as potential diagnostic biomarkers and that lipid metabolism plays a role in the pathogenesis of AIS.

Adolescent idiopathic scoliosis (AIS) is a complex three dimensional deformity of the spine, characterized with lateral curvature in the coronal plane and vertebral rotation in the transverse plane. AIS is the most common form of scoliosis and affects approximately 1–3% of children aged between 10 and 16 years^1^. Severe scoliosis can progress to chest and rib deformity, leading to cardiac and respiratory compromises, and can also lead to cosmetic problems and emotional distress for some patients^2^. Adobor *et al.*^3^ reported that the mean major curve of idiopathic scoliosis patients at first presentation was 38° without screening, approaching the upper limit for indications for conservative treatment with bracing. Earlier diagnosis increases the opportunity for successful conservative treatment and hence reduces the need for surgical intervention^4^,^5^.

Currently, the diagnosis and screening of AIS is heavily dependent on the clinical asymmetry appearances of patients and radiographic images^6^,^7^. Scoliometer used along with an Adams Forward Bend Test is considered as the best tool to measure trunkal asymmetry for scoliosis screening^5^. However, screening for scoliosis is still controversial. The United States Preventive Services Task Force and American Academy of Family Physicians recently recommended against routine scoliosis screening in asymptomatic adolescents, because its low specificity would expose many low-risk adolescents to unnecessary radiographs and referrals^8^. Therefore there is a pressing need to find new diagnostic biomarkers to facilitate accurate early detection^6^,^7^.

AIS is thought to be a multifactorial disorder, involving genetic factors, nervous system, hormones and metabolic dysfunction, skeletal growth, biomechanical factors and environmental and lifestyle factors^9^. Despite considerable efforts, the etiopathogenesis of AIS remained largely unknown. Previous studies have investigated several proteins related to AIS. However, their application prospects as diagnostic biomarkers were poor or not fully evaluated^10^,^11^,^12^,^13^,^14^. Platelet calmodulin levels were found to correlate closely with curve progression and stabilization by bracing or spinal fusion^10^. However, the lack of normal data and the large variability in the baseline levels limited its potential use and necessitated the use of the AIS patients as their own control^11^. Qiu *et al.* in 2007 12, first reported decreased leptin levels in AIS patients. Yet, later the same research group found no significant differences in total leptin levels between AIS females and healthy controls, but significant differences in the ratio of leptin to soluble leptin receptor^13^. Recently, abnormal levels of plasma osteopontin, soluble CD44 and serum ghrelin were found in some AIS patients^14^. However, further research and a validated method for early diagnosis were still needed.

The advent and development of metabonomics enables researchers to detect a large number of small-molecule metabolites quantitatively in a single step^15^, providing immense potentials in discovering disease-related biomarkers. Metabolic profiles often reflect the consequences of the pathophysiological process and may assist the development of novel diagnostic tests. Thus, we conducted this serum metabonomic study on AIS patients using ultra high-performance liquid chromatography coupled with quadrupole time-of-flight mass spectrometry (UPLC/QTOF-MS). A two-stage study design was utilized—discovery stage and replication stage. The primary aim was to discover potential diagnostic biomarkers of AIS, and the secondary aim was to explore the mechanism of the “presumed abnormal metabolic profiles” of AIS.

## Results

### Demographic data of AIS patients and healthy controls

Demographic data and biochemical index of all participants are shown in Table 1. The proportion of females was 82.8% in the whole population as AIS affected females more frequently. There were no significant difference of age and sex ratio between both groups in the discovery and replication sets (*P* > 0.05). The mean weight between the two groups was also similar in both sets (*P* > 0.05). The Cobb angles of the main curve, which represented the severity of scoliosis, ranged from 30° to 92° in the discovery set, and 30° to 78° in the replication set, suggesting moderate to severe scoliosis of the collected cohorts. Common biochemical indices, including alamine aminotransferase, total bilirubin, direct bilirubin, creatinine and urea, also showed no statistical significance between the two groups in both sets (*P* > 0.05).

### Serum metabolic profiles of AIS patients in discovery set

**Figure 1:**
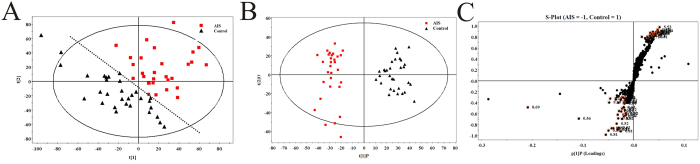
PCA and OPLS-DA based on UPLC/QTOF-MS data of serum samples obtained from AIS and healthy controls in discovery set. (**A**) PCA score plot; (**B**) OPLS-DA score plot; (**C**) S-plot.

The metabolic profiles of serum samples from 30 AIS patients and 31 healthy controls in the discovery set were acquired using UPLC/QTOF-MS in positive mode. The representative base peak intensity chromatograms from both groups are illustrated in Supplementary Figure S1. The PCA analysis was performed by the Markerlynx 4.1 software to discern the metabolic profiles of AIS and healthy controls. The score plot showed that the metabolic profiles of AIS patients generally deviated from the healthy controls, suggesting that significant biochemical changes occurred in AIS patients (Fig. 1A).

An OPLS-DA model was employed to sharpen the already established separation by PCA. As expected, superior separation of AIS and controls was observed in score plot of OPLS-DA (Fig. 1B). The corresponding S-plot indicated that the ions with high variable importance (VIP ≥1) were responsible for discriminating patients and healthy participants (Fig. 1C, Table 2).

**Figure 2:**
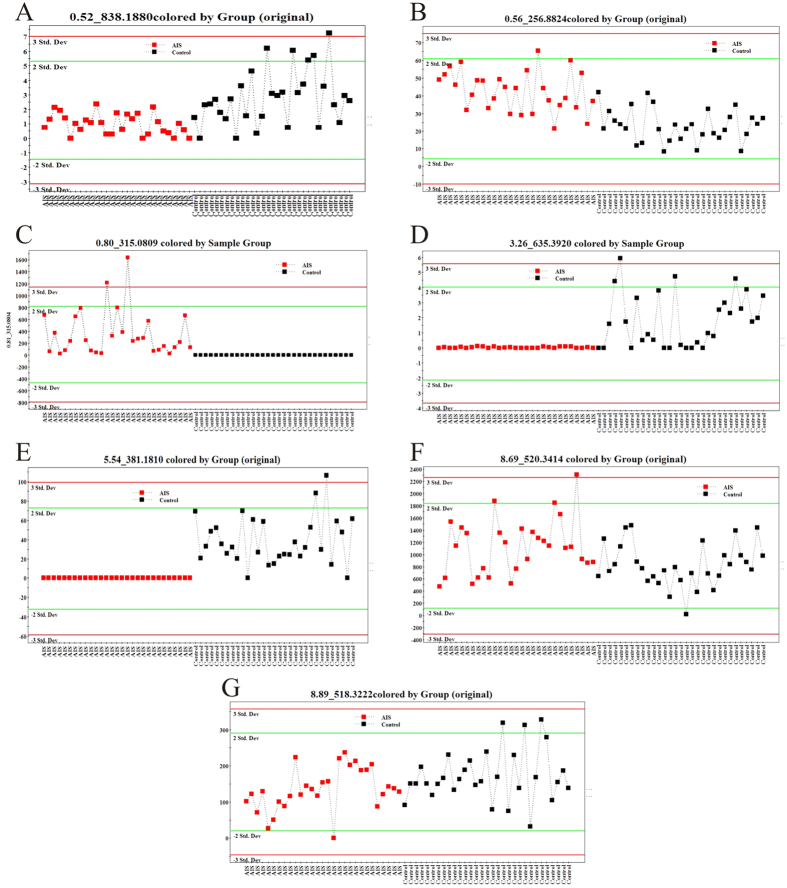
Concentrations of the differential metabolites between AIS and healthy controls in discovery set. (**A**) PC(40:4), Retention time (RT): 0.52, m/z: 838.188; (**B**) 2-Hexenoylcarnitine, RT: 0.56, m/z: 256.8824; (**C**) Beta-D-Glucopyranuronic acid, RT: 0.80, m/z: 315.0809; (**D**) DG(38:9), RT: 3.26, m/z: 635.3920; (**E**) MG(20:3), RT: 5.54, m/z: 381.1810; (**F**) LysoPC(18:2), RT: 8.69, m/z: 520.3414; (**G**) LysoPC(16:0), RT: 8.89, m/z: 518.3222.

The structures elucidation was carried out by analyzing their accurate molecular weights and MS^E^ spectra from HMDB (http://www.hmdb.ca/) and KEGG (http://www.genome.jp/kegg/) within a mass difference lower than 5 ppm. As a result, seven “differential metabolites” were identified, including: PC(40:4) (**1**), 2-hexenoylcarnitine (**2**), beta-D-glucopyranuronic acid (**3**), DG(38:9) (**4**), MG(20:3) (**5**), LysoPC(18:2) (**6**) and LysoPC(16:0) (**7**) (Table 2). As showed in the trends plot (Fig. 2), the levels of PC(40:4) (**1**), DG(38:9) (**4**), MG(20:3)(**5**), LysoPC(18:2) (**6**) and LysoPC(16:0) (**7**) were significantly decreased in AIS patients compared with the control group. Meanwhile, the levels of 2-hexenoylcarnitine (**2**) and beta-D-glucopyranuronic acid (**3**) were significantly increased in AIS group.

### Validation of diagnostic biomarkers in replication set through UPLC/QTOF-MS

**Figure 3:**
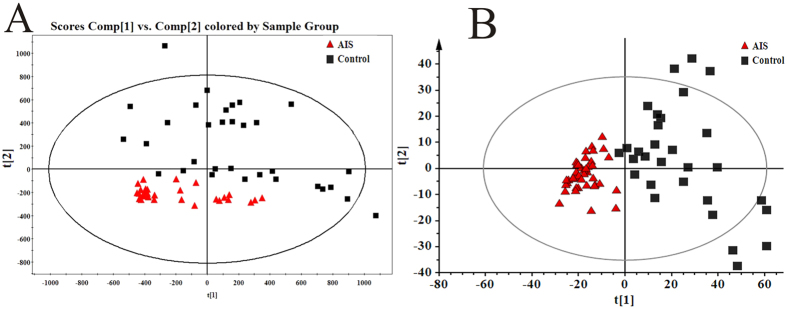
PCA score plot in replication set. (**A**) PCA score plot based on all metabolites; (**B**) PCA score plot based on six potential biomarkers.

To validate the findings in discovery set, serum samples of 31 AIS patients and 44 healthy controls were collected and analyzed with the same analytical procedures as the discovery set. The metabolic profiles of AIS samples also deviated from healthy controls indicated by PCA analysis (Fig. 3A), mainly in [t2] axis, suggesting favorable repeatability of UPLC-MS assays. To validate the values of the potential diagnostic biomarkers for accurate diagnosis, only the seven differential metabolites identified in discovery stage were used as variables and imported into SIMCA-P software. A new score plot of AIS and control by PCA analysis was performed. As showed in Fig. 3B, the metabolite profiles of AIS and normal were clearly discriminated from each other in the [t1] axis, which meant superior separation than that in [t2] axis. In addition, the Q2 and R2 values of the new PCA model were 0.786 and 0.928, which was higher than the PCA model containing all the variables (Q2 = 0.14, R2 = 0.26), suggesting greatly improved capacities of model prediction.

**Figure 4:**
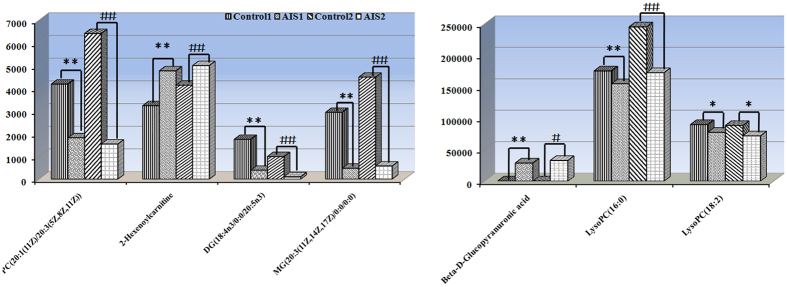
Levels of the seven potential biomarkers with diagnostic values in both discovery set and replication set (**P < 0.01 in discovery set; ^##^P < 0.01 in replication set).

Mean peak areas of the seven candidate diagnostic biomarkers between AIS patients and healthy participants were compared in discovery set and replication set, respectively. It turned out that the seven potential diagnostic biomarkers were significantly different in serum samples of AIS to the same tendencies in both discovery and replication sets (*P* < 0.01; Fig. 4), which meant the concentrations of these metabolites in AIS patients were relatively stable. This was valuable for the use of diagnosis of AIS. However, as illustrated in Fig. 4, changes of these metabolites between the two sets of samples could still observed. Qualification and validation with a larger number of samples is still needed.

Correlations between these seven candidate diagnostic biomarkers and main clinical features were analyzed in the supplementary information (Table S1). Stratification of all AIS patients based on the severity of the Cobb angle were performed and PCA analysis was proceeded (Supplementary Figure S2).

### Expressions of lecithin: cholesterol acyltransferase (LCAT) in serum and adipose triglyceride lipase (ATGL) and hormone sensitive lipase (HSL) in adipose tissue

**Figure 5:**
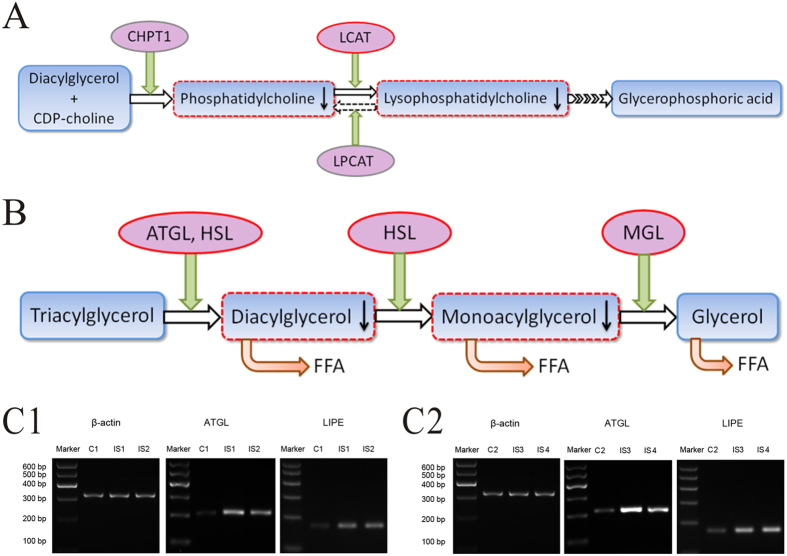
Metabolic pathway related to potential biomarkers indicated by UPLC-MS analysis and RT-PCR results of ATGL and HSL. (**A**) metabolic pathway of LysoPC; (**B**) catabolism pathway of triacylglycerol; C1-C2, RT-PCR results of ATGL and HSL in adipose tissues (LIPE is the gene aliase of HSL; C1-2, adipose tissues from the two control patients; IS1-4, adipose tissues from the four AIS patients). CHPT1, choline phosphotransferase 1; LCAT, lecithin: cholesterol acyltransferase; LPCAT, lysophosphatidylcholine acyltransferase; ATGL, adipose triglyceride lipase; HSL, hormone sensitive lipase; MGL, monoacylglycerol lipase; FFA, free fatty acid.

The mechanism of disturbed metabolic pathways of AIS was further explored. LCAT is a serum enzyme that catalyzes the transacylation of the sn-2 fatty acid of phosphatidylcholine to the free 3-hydroxyl group of cholesterol, generating lysophosphatidylcholine (LysoPC) and cholesteryl esters^16^. LysoPC has a role in lipid signaling by acting on LysoPC receptors. In blood, significant amounts of LysoPC are formed by this specific enzyme system. Two types of LysoPC (LysoPC(18:2) (**6**) and LysoPC(16:0) (**7**)) were significantly decreased in serum samples of AIS patients. Thus, detection of serum LCAT activity was performed using fluorescent ELISA (Fig. 5A). The mean ratio of the two emission intensities (460 nm/405 nm), which reflected LCAT activity, were 1.117 ± 0.048 and 1.122 ± 0.05 in AIS patients and healthy participants, respectively. No statistical significance was observed (*P* = 0.812), indicating that differential LysoPCs of AIS were not caused by LCAT.

Decreased levels of diacylglycerol (DG(38:9), (**4)**) and monoacylglycerol (MG(20:3), (**5)**), which were decomposition products of triacylglycerol (TG), were observed in present study (Fig. 5B). The breakdown of stored TG is largely regulated by ATGL and HSL^17^. mRNA expression levels of ATGL and HSL were analyzed by Reverse transcription-polymerase chain reaction (RT-PCR). Compared with healthy participants, band intensities of ATGL and HSL in AIS patients were significantly increased, suggesting higher expressions of ATGL and HSL in adipose tissues of AIS patients (Fig. 5C).

## Discussion

Early diagnosis of AIS could potentially reduce surgical intervention, however clinically useful and accurate diagnostic biomarkers have yet to be realized. Metabonomic study enabled us to discover diagnostic biomarkers through an untargeted metabolites searching approach. Using metabolic profiling, seven differential metabolites characterizing AIS were identified by the OPLS-DA model in discovery set. To the best of our knowledge, all the differential metabolites were firstly identified as potential biomarkers in serum samples of AIS. The discrimination power of these seven diagnostic biomarkers was validated by UPLC/QTOF-MS analysis in replication set, thus minimizing false-positive findings.

Of the seven differential metabolites, six were categorized as lipids; the remaining was one type of glucuronic acid derivatives (beta-D-glucopyranuronic acid (**3**)). The six types of lipids were composed of three kinds of glycerophospholipids (PC(40:4) (**1**), LysoPC(18:2) (**6**) and LysoPC(16:0) (**7**)), two kinds of glycerolipids (DG(38:9) (**4**) and MG(20:3) (**5**)) and one kind of fatty acid esters (2-hexenoylcarnitine (**2**)) (Table 2). These lipids were involved in glycerophospholipid metabolism, glycerolipid metabolism and fatty acid metabolism, suggesting significantly perturbed lipid metabolism occurred in AIS (Fig. 5A,B).

Glycerophospholipids are important membrane lipids and play a vital role in cellular functions, such as signal transductions, regulations of transport processes and protein functions^18^. In addition, glycerophospholipids are essential components of lipoproteins and influence their metabolism and functions^19^,^20^. In a recent metabonomic study, abnormal glycerophospholipid metabolism has been detected in murine osteoclast treated by estradiol^21^. Differentiated LysoPCs were also observed in plasma samples of the osteoporotic rat’s model^22^. These results are consistent with osteopenia and deranged bone quality presenting in AIS^23^,^24^. Interestingly, lower estrogen content or their abnormal action on respective target cells, e.g. bone cells, are supposed to be one of the etiologic factors of AIS^25^,^26^. Estrogens interact with many pathophysiological factors that are believed to influence the development of scoliosis, such as modulation of growth factors, inhibition of melatonin synthesis, interaction with calmodulin, exacerbation of response to strain and bone formation/resorption^26^. We detected the perturbed glycerophospholipid pathway in AIS patients, which might be caused by abnormal biological functions of estrogens of AIS. Thus, PCA analysis was further performed between males and females in AIS patients and healthy controls. However, the metabolic profiles were not generally deviated in both AIS patients and controls (supplementary Figure S3). Other mechanisms, which were not caused by sex, might exist between in AIS. LCAT is a key enzyme in the process of LysoPC metabolism (Fig. 5A). However, we did not observe abnormal LCAT activity in AIS patients, suggesting the decreased LysoPCs were probably caused by other mechanisms.

Abnormal glycerolipid degradation was largely regulated by ATGL and HSL, which aroused our interest in exploring their expressions (Fig. 5B). Because ATGL and HSL are mainly located in and play a role in adipose tissue, mRNA expression levels in adipose tissue were assayed by RT-PCR. Increased expressions of ATGL and HSL were observed in AIS patients, suggesting increased lipolysis of AIS patients. Additionally, increased 2-hexenoylcarnitine (**2**), a fatty acylcarnitine, was observed in AIS patients. Fatty acylcarnitines act as the medium which assist the transportation of long chain fatty acid into mitochondria and increased serum fatty acylcarnitines concentrations are reported to reflect long chain fatty acid *β*-oxidation, thus indicating increased lipid metabolism^27^,^28^,^29^. These were consistent with previous reports of lower body mass index and low fat mass in AIS compared with general population^30^,^31^. In fact, it has already been postulated that AIS has a dysfunctional energy balance involving a complex systems including white adipose tissue, the adipose-tissue derived hormone leptin and other cytokine-hormones, hypothalamus and neuroendocrine axes^32^.

Controversies were still existed about the pathogenesis of AIS. However, researchers reached a consensus on its multifactorial etiologies. Hormones and metabolic dysfunction was thought to be one of the top theories^9^. In this study, perturbed glycerophospholipid metabolism, glycerolipid metabolism and fatty acid metabolism were firstly discovered in AIS patients, validating the postulation of disturbed energy metabolism of AIS by some researchers^30^,^32^. Energy homeostasis are regulated by integratory centers in the central nervous system which receive and convey signals from peripheral organs and then send efferent neural and hormonal signals to peripheral tissues. So we postulated that the perturbed lipid metabolism in AIS could be the manifestation of an abnormal neuroendocrine system, caused by genetic variations^9^,^33^,^34^.

There were some limitations in the current study. Firstly, serum samples of AIS in both discovery and replication set came from patients seeking surgical intervention, which represented relatively severe scoliotic deformities. So the results might not be extrapolated to AIS patients who present at an earlier stage. The early diagnostic values of the potential biomarkers discovered in our study still need to be further validated in larger clinical trials. In addition, given the nature of the cross-sectional study design, the differentiated metabolic profiles of AIS might be confused by other confounding factors, such as different dietary intakes. However, all the serum samples were collected at the same time (fasting morning) in both cases and controls. Most importantly, a two-stage study design was used and the differential metabolites discovered in the first cohort were further confirmed in an independent population-based replication sample, thus enhancing the reliability of our results. Finally, RT-PCR assays of ATGL and HSL were not analyzed quantitatively due to the small number of adipose tissue samples collected. Further larger studies focusing on the pathogenesis of AIS are required to confirm this.

## Conclusions

Our study has provided serum characteristic metabolic profiles of AIS patients. Seven differential metabolites were identified from metabonomic analysis in the discovery set. These candidate diagnostic biomarkers were validated by metabonomic analysis in an independent replication set. Differential metabolites suggested a disrupted lipid metabolism in AIS, including glycerophospholipid metabolism, glycerolipid metabolism and fatty acid metabolism. Additionally, related proteins of the perturbed pathways showed elevated ATGL and HSL in adipose tissues of AIS, providing clues for further researches of the pathogenesis of AIS. We believe that our study is one further step closer to finding a clinically useful and validated diagnostic biomarker test for early detection of scoliosis which could potentially reduce the need for invasive surgical correction.

## Methods

### Subjects and sample collection

Both discovery samples and replication samples were derived from patients seeking surgical treatment in Peking Union Medical College Hospital. They were age and sex matched with healthy controls. 30 AIS cases and 31 healthy controls were selected at discovery stage whilst 31 AIS cases and 44 healthy controls at replication set. The diagnosis of AIS was made pre-operatively by experienced surgeons mainly based on rotational rib prominence during the Adams Forward Bend Test and a maximum Cobb angle above 10°. All other types of scoliosis, such as syndromic scoliosis and congenital scoliosis, were excluded from our study. Meanwhile, the healthy controls were firstly tested with Adams Forward Bending Test to rule out any scoliosis. In the case of any uncertainty, radiographs were performed for validation.

Fasting blood samples were collected in AIS patients preoperatively as well as in healthy controls. Collected blood samples were left to clot for two hours at room temperature, then centrifuged at 3000 rpm for 15 minutes at 4 °C. Serum was aliquoted and stored at −80 °C until use in the assay.

To assay mRNA levels of ATGL and HSL, subcutaneous adipose tissues of 4 AIS patients and 2 controls (one from a 17 year-old female patient with a diagnosis of genu valgum and the other from a 16 year-old female patient with gluteus contracture) were collected intra-operatively.

The study was approved by the institutional review board of Peking Union Medical College Hospital and written informed consents were obtained from all participants. All experimental procedures were carried out in accordance with the approved guidelines.

### Serum Metaobnomics study by UPLC/QTOF-MS

#### Sample preparation

The metabolites extraction process was performed according to procedures outlined in our previously published study with minor modifications^35^. Briefly, 200 μL serum was added into 800 μL methanol-acetonitrile mixture (4:1, v/v), vortex-mixing undertaken for 1 minute. After 5 minutes standing in ice-bath, the above mixture was centrifuged at 13000 rpm for 15 minutes at 4 °C to precipitate the proteins. Supernatant was collected and dried with nitrogen at 37 °C. The dried residue was reconstituted in 200 μL 30% (by volume) acetonitrile in water, then vortex-mix for 1 minute. After centrifugation at 13000 rpm for a further 15 minutes at 4 °C, 2 μL of supernatant was injected for UPLC/QTOF-MS analysis.

#### Data acquisition

Chromatographic separation was preceded on an Acquity UPLC HSS T3 column (2.1 mm ×100 mm, 1.8 μm, Waters Corp., Milford, USA) using Waters Acquity^TM^ UPLC system. The column was maintained at 40 °C and eluted at a flowing rate of 0.45 mL/min, using a mobile phase of (A) 5% (by volume) acetonitrile in water and (B) 95% (by volume) acetonitrile in water. The gradient program was optimized as follows: 0–5 min, 1%B to 45%B; 5–9 min, 45%B to 70%B; 9–11 min, 70%B to 99%B; 11–13 min, washing with 99%B, and 13–17 min, equilibration with 1%B. The eluent from the column was directed to the mass spectrometer without split.

A Waters SYNAPY G2 HDMS (Waters Corp., Manchester, UK) was used to perform the mass spectrometry with an electrospray ionization source operating in positive ion mode. The capillary voltage was set to 3.0 kV. Sample cone voltage and extraction cone voltage were at 40 V and 4 V, respectively. Using drying gas nitrogen, the desolvation gas rate was set at 800 L/h at 450 °C, the cone gas rate at 50 L/h, and the source temperature at 120 °C. The scan time and inter scan delay were set at 0.3 s and 0.02 s, respectively. Leucine-enkephalin was used as the lockmass in positive ion mode (*m/z* 556.2771[M + H]^+^). Data was collected in centroid mode from *m/z* 50–1200 Da.

To validate the stability of sequence analysis, 10 μL samples were extracted from randomly selected 10 AIS patients and 10 controls and were pooled as a quality control sample (QC). The pooled QC sample was prepared in the same way as the other samples and analyzed randomly through the analytical batch. The extracted ion chromatographic peaks of ten ions in positive mode were selected for method validation, as retention time (RT) and *m/z* pairs of 0.89_315.0804, 1.47_100.076, 2.77_459.2798, 6.39_274.2743, 7.79_302.3052, 8.45_520.3411, 8.69_520.3419, 9.18_496.3415, 9.61_522.3569 and 10.57_524.3725. The relative standard deviations (RSD%) of RT and *m/z* were 0–1.79% and 0.01–0.13%, respectively. The repeatability of method was assessed using six replicates of QC sample in positive ion mode. The RSD% of RT and *m/z* were 0–2.37% and 0.02–0.12%, respectively.

#### Multivariate statistical analysis

The raw spectral data were first analyzed with MassLynx Applications Manager Version 4.1 (Waters, Manchester, UK). Deconvolution, alignment and data reduction were performed to provide a list of RT and mass pairs with corresponding peak area for all the detected peaks from each file in the data set. The main parameters were set as follows: RT range 0.5–14 min; mass range 50–1200; XIC window, 0.02 Da; automatically calculate peak width and peak–peak base-line noise; use the raw data during the deconvolution procedure; marker intensity threshold (count), 300; mass tolerance, 0.02 Da; RT windows, 0.2 s; noise elimination level, 6; retain the isotopic peaks.

The resulting UPLC-MS data were then transferred to SIMCA-P software package (version 12.0, Umetric, Umeå, Sweden). Principal component analysis (PCA), which mapped samples based on their spectral profile without using previous knowledge of class, was used to explore inherent grouping between AIS patients and healthy controls by visual inspection of score plots. Supervised models were subsequently performed by orthogonal partial least squares discriminant analysis (OPLS-DA) to maximize the separation between different classes and identify biomarkers associated with AIS. The results were visualized in the form of score plots and potential biomarkers were selected on the basis of variable importance in the project (VIP) value and S-plot.

#### Fluorescent enzyme-linked immune sorbent assay of LCAT activity

LCAT activity was assayed with LCAT kit as per manufacturer’s instructions in serum samples of 12 AIS patients and 12 healthy controls (Calbiochem/EMD-Millipore/Merck KGaA, Darmstadt, Germany). Each sample was tested in duplicate. Briefly, 1 μL LCAT substrate was mixed with 200 μL LCAT assay buffer containing 5 μL serum samples. The mixture was then incubated for 4 hours at 37 °C. Then, 100 μL of the mixture was added to 300 μL READ reagent. The fluorescence at an excitation wavelength of 355 nm and emission wavelengths of 405 nm and 460 nm was read. The ratio of the two emission intensities at 460 nm and 405 nm were analyzed between the two groups.

#### RT-PCR for ATGL and HSL in adipose tissue

Total RNA was extracted using Trizol Reagent (Life Technologies AB & Invitrogen, Carlsbad, USA) and then converted into cDNA with RevertAid First Strand cDNA Synthesis Kit (Thermo Fisher Scientific Inc., Waltham, USA). Using β-actin as an internal reference, PCR was preceded using FastStart Universal SYBR Green Master (F. Hoffmann-La Roche Ltd., Basel, Switzerland) (Supplementary Table S2). All the procedures were performed following the manufacturer’s instructions. Agarose gel electrophoresis was then performed and fluorescent strips were recorded.

#### Statistical analysis

Statistical analysis was conducted using SPSS software version 16.0 (Chicago, IL, USA). All the numerical variables between AIS patients and healthy participants, including age, weight, biochemical indexes, LCAT emission intensity and mean peak areas of representative metabolites, were compared by two-tailed Student’s t-test. Sex ratio was compared using Fisher’s exact test. *P* values less than 0.05 were set as statistically significant.

## Additional Information

**How to cite this article**: Sun, Z.-j. *et al.* Identification of candidate diagnostic biomarkers for adolescent idiopathic scoliosis using UPLC/QTOF-MS analysis: a first report of lipid metabolism profiles. *Sci. Rep.* **6**, 22274; doi: 10.1038/srep22274 (2016).

## Supplementary Material

Supplementary Information

## Figures and Tables

**Table 1:** Baseline data of AIS patients and healthy participants.

Characteristics	Discovery set			Replication set		
	AIS patients	Healthy controls	*P*	AIS patients	Healthy controls	*P*
Sex ratio (male: female)	4:26	0:31	0.053	3:28	11:33	0.134
Age (years ± SD)[range]	13.9 ± 1.5 (12–17)	13.5 ± 2.5 (8–16)	0.525	14 ± 2.1(11–18)	14.1 ± 2(10–16)	0.856
Weight (kilogram ± SD) [range]	50.2 ± 11(35–83)	47.5 ± 13.7(24.5–96)	0.427	46.8 ± 10.9(35–82)	50 ± 7.7(41–69)	0.257
Cobb angle of main curve^a^ (degree ± SD)[range]	48.9 ± 13.3 (30–92)	None	–	48.1 ± 12.3(30–78)	None	–
Biochemical index						
ALT (U/L ± SD) [range]	12.5 ± 5.6(6–32)	14.8 ± ± 9.1(5–49)	0.348	11.8 ± 7.2(6–34)	16.1 ± 13.6(4–46)	0.278
TBil (μmol/L ± SD) [range]	9.7 ± 4.2(3.8–19)	10.1 ± 5.8(3–26)	0.821	9.6 ± 4.7(4.9–23.8)	9.7 ± 4.8(4.1–20.3)	0.927
DBil (μmol/L ± SD) [range]	3.6 ± 1.7(1.4–7.8)	3.4 ± 1.9(1.4–8.4)	0.696	3.5 ± 1.5(1.6–9.2)	4 ± 2.1(2.2–8.4)	0.441
Cr (μmol/L ± SD) [range]	53 ± 7.7(39–69)	52.5 ± 13.6(32–85)	0.893	54.6 ± 10(36-75)	60.9 ± 14.4(40–94)	0.129
Urea(mmol/L ± SD) [range]	4.6 ± 0.9(3–6.3)	4.9 ± 2.2(2.2–12.2)	0.629	4.4 ± 1.1(2.9–7.5)	4.1 ± 0.9(2.9–5.9)	0.335

AIS, adolescent idiopathic scoliosis; SD, standard deviation; ALT, alamine aminotransferase; TBil, total bilirubin; DBil, direct bilirubin; Cr, creatinine.

^a^Cobb angle of main curve was a primary parameter to identify the severity of scoliosis.

**Table 2:** Serum differential metabolites detected by UPLC/QTOF-MS of AIS patients.

No.	metabolite	RT (min)	m/z	Main fragment	formula	Tendency in AIS	VIP	Pathway
S1	PC(40:4)	0.52	838.188	446.8888	C_48_H_88_NO_8_P	↓	1.98	Glycerophospholipid metabolism
				378.9026				
				258.899				
				226.9522				
S2	2-Hexenoylcarnitine	0.56	256.8824	191.040	C_13_H_23_NO4	↑	17.4	Camitine synthesis
				162.113				
				118.0865				
S3	Beta-D-Glucopyranuronic acid	0.80	315.0809	293.0620	C_13_H_14_O_9_	↑	5.03	Glucuronidation
				160.0907				
				132.0655				
S4	DG(38:9)	3.26	635.3920	657.3838	C_41_H_62_O_5_	↓	4.19	Glycerolipid metabolism
S5	MG(20:3)	5.54	381.1810	133.0848	C_23_H_40_O_4_	↓	8.79	Glycerolipid metabolism
S6	LysoPC(18:2)	8.69	520.3414	542.3232	C_26_H_50_NO_7_P	↓	33.38	Glycerophospholipid metabolism
				483.2481				
				184.0735				
S7	LysoPC(16:0)	8.89	518.3222	496.3406	C24H50NO7P	↓	5.24	Glycerophospholipid metabolism
				459.2487				

RT, retention time; VIP, variable importance in the projection; PC, phosphatidylcholine; DG, diacylglycerol; MG, monoacylglycerol; LysoPC, lysophosphatidylcholine.
