# Supplementary material for: Identification of candidate diagnostic biomarkers for adolescent idiopathic scoliosis using UPLC/QTOF-MS analysis: a first report of lipid metabolism profiles
Source: Sci Rep. 2016 Mar 1;6:22274. doi: 10.1038/srep22274 (PMC4772092; doi:10.1038/srep22274)
Supplement: Supplementary Information [file srep22274-s1.doc]

**Identification of** **candidate diagnostic biomarkers for** **adolescent idiopathic scoliosis using UPLC/QTOF-MS analysis: a first report of lipid metabolism profiles**

Zhi-jian Sun^1^, Hong-mei Jia^2^, Gui-xing Qiu^1^, Chao Zhou^2^, Shigong Guo^3^, Jian-guo Zhang^1^, Jian-xiong Shen^1^, Yu Zhao^1^,Zhong-mei Zou^2^

1. Department of Orthopaedics, Peking Union Medical College Hospital, Chinese Academy of Medical Sciences and Peking Union Medical College, Dongcheng District Shuaifuyuan No.1, Beijing 100730, China
2. Institute of Medicinal Plant Development, Chinese Academy of Medical Sciences and Peking Union Medical College, No. 151 Malianwa North Road, Haidian District, Beijing 100193, China
3. Department of Trauma &Orthopaedic Surgery, Lister Hospital, Stevenage, UK


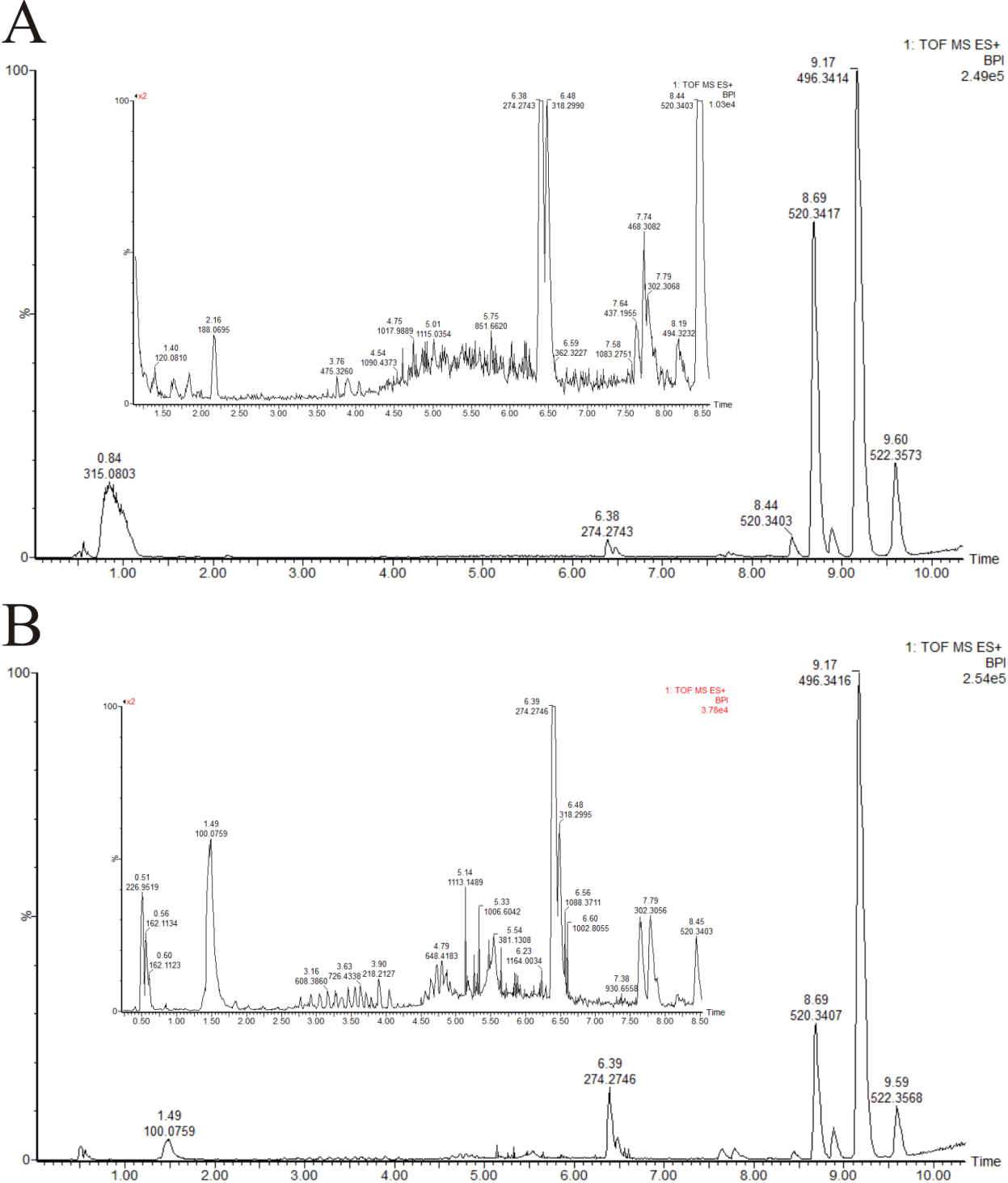


Figure S1 Base peak intensity chromatograms of AIS (A) and controls (B).

**Correlations of the seven diagnostic biomarkers and main clinical features**

AIS patients in both discovery set and replication set were further stratified according to the relative levels (mean peak areas) of the seven potential diagnostic biomarkers. They were divided into two groups based on the mean peak areas of each biomarkers (the lower half group and the upper half group), respectively. The mean ages were significantly different when divided by the relative levels of PC(40:4) and Beta-D-Glucopyranuronic acid, which meant levels of PC(40:4) and Beta-D-Glucopyranuronic acid might be correlated with age (Table S1). Pearson correlation analysis between levels of PC(40:4) and Beta-D-Glucopyranuronic acid and age were further performed. Significant linear correlation between PC(40:4) levels and age in AIS patients were identified (*P*=0.014); however, significant correlation was not existed between Beta-D-Glucopyranuronic acid levels and age (*P*=0.454).

Table S1 Correlations of the diagnostic biomarkers and main clinical features in AIS patients

| **characters** | **PC(40:4)** | | | **2-Hexenoylcarnitine** | | | **Beta-D-Glucopyranuronic acid** | | | **DG(38:9)** | | | **MG(20:3)** | | | **LysoPC(18:2)** | | | **LysoPC(16:0)** | | |
| --- | --- | --- | --- | --- | --- | --- | --- | --- | --- | --- | --- | --- | --- | --- | --- | --- | --- | --- | --- | --- | --- |
|  | Lower | Upper | P | Lower | Upper | P | Lower | Upper | P | Lower | Upper | P | Lower | Upper | P | Lower | Upper | P | Lower | Upper | P |
| **Sex ratio (male : female)** | 2:29 | 5:25 | 0.241 | 4:27 | 3:27 | 1.000 | 4:27 | 3:27 | 0.707 | 3:28 | 4:26 | 1.000 | 4:27 | 3:27 | 1.000 | 3:28 | 3:27 | 1.000 | 4:28 | 3:26 | 1.000 |
| **Age (years±SD)** | 13.3±1.3 | 14.6±2.1 | **0.006** | 14.1±1.8 | 13.8±1.9 | 0.993 | 13.7±1.4 | 14.1±2.2 | **0.013** | 13.5±1.7 | 14.4±1.8 | 0.903 | 13.5±1.6 | 14.4±2.0 | 0.205 | 13.5±1.6 | 14.4±2 | 0.072 | 13.7±1.7 | 14.2±1.9 | 0.865 |
| **Cobb angle of main curve**  **(degree±SD)** | 46.2±12.1 | 51.6±13.1 | 0.891 | 49.0±13.7 | 48.8±12.1 | 0.326 | 47.3±11.2 | 50.4±14.3 | 0.324 | 49.4±13.9 | 48.3±11.8 | 0.593 | 48.9±13.7 | 48.8±12.1 | 0.436 | 48.9±13.7 | 48.8±12.1 | 0.984 | 49.0±10.3 | 48.7±15.1 | 0.114 |
| **Correction rate (%)^a^** | 80.5±10.3 | 82.4±11.7 | 0.321 | 82.4±10.7 | 80.4±11.3 | 0.427 | 80.3±9.4 | 82.6±12.4 | 0.056 | 82.0±10.9 | 80.9±11.2 | 0.819 | 80.4±10.6 | 82.4±11.4 | 0.770 | 80.4±10.6 | 82.4±11.4 | 0.499 | 85.1±10.9 | 77.8±10.0 | 0.946 |
| **Fusion level** | 9.5±2.6 | 10.1±2.6 | 0.821 | 9.7±2.4 | 10.0±2.8 | 0.283 | 10.0±2.4 | 9.8±2.9 | 0.311 | 10.4±2.7 | 9.3±2.4 | 0.288 | 9.7±2.5 | 9.9±2.7 | 0.663 | 9.7±6.7 | 9.1±7.9 | 0.775 | 9.8±2.5 | 9.9±2.8 | 0.505 |

a Correction rate was calculated by (preoperative angle－postoperative angle)/preoperative angle×100%.

**PCA analysis based on the severity of AIS**

Stratification of all AIS patients in both discovery set and replication set were performed based on the severity of the Cobb angle. AIS patients were divided into three groups: mild (the main Cobb angle <40°), moderate (the main Cobb angle >40°and <50°) and severe (the main Cobb angle >50°). PCA analysis was preceded. However, no generalized separations were observed (Figure S2), suggesting the metabolic profiles of AIS patients might be not severity-dependent.


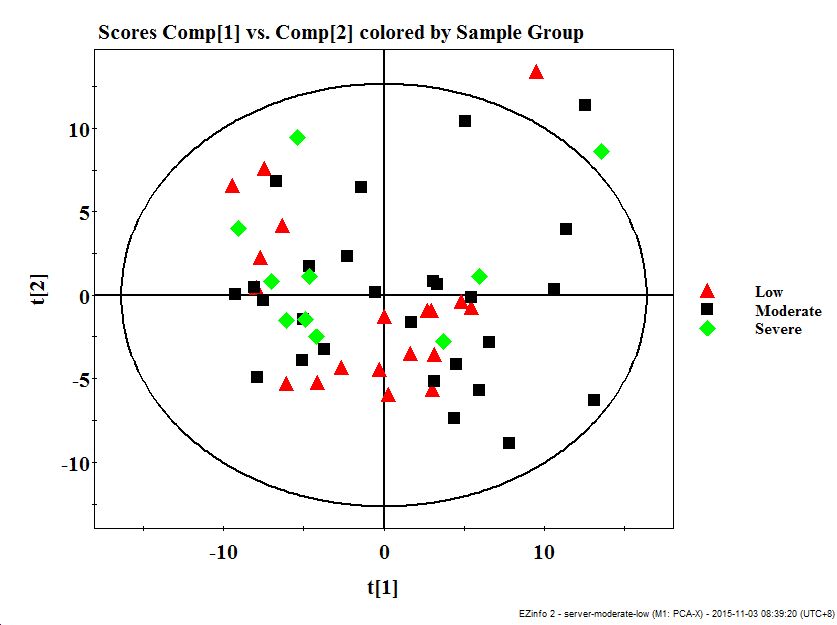


Figure S2 PCA score plot of all AIS patients divided by the severity of Cobb angle.


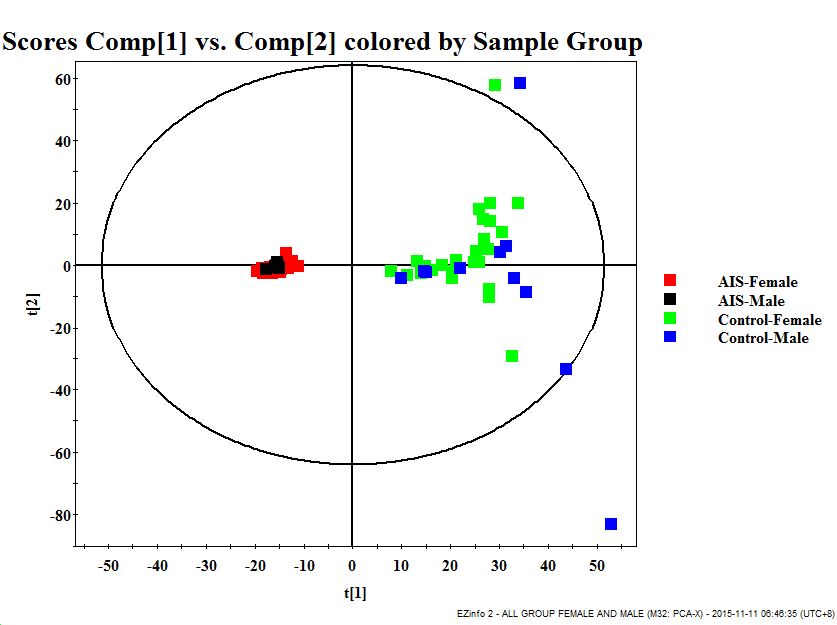


Figure S3 PCA score plot between males and females of all AIS patients and healthy controls in both sets.

Table S2 List of primers in RT-PCR

| Accession number | Primers’ name | Sequences (5’-3’) | Fragment length (bp) |
| --- | --- | --- | --- |
| [NM_001101](http://www.ncbi.nlm.nih.gov/nuccore/168480144) | H-ACTIN-S  H-ACTIN-A | CACCCAGCACAATGAAGATCAAGAT  CCAGTTTTTAAATCCTGAGTCAAGC | 317 |
| [NM_020376.3](http://www.ncbi.nlm.nih.gov/entrez/viewer.fcgi?db=nucleotide&id=300796746) | H-ATGL-S  H-ATGL-A | TGTCAGACGGCGAGAATGTCAT  CCTGCGGACAGATGTCACTCTC | 228 |
| [NM_005357.3](http://www.ncbi.nlm.nih.gov/entrez/viewer.fcgi?db=nucleotide&id=542133076) | H-LIPE-S^a^  H-LIPE-A | ACTCTTCTTTGAGGGCGACGA  GTTTGTAGTGCTCCCCGAAGG | 173 |

a LIPE is the gene aliase of HSL.
